# The Relevance of Lymphadenectomy Extension to the Right Paratracheal Space in the Treatment of Esophagogastric Junction Adenocarcinoma: A Retrospective Bicentric Study

**DOI:** 10.3390/curroncol32110609

**Published:** 2025-10-31

**Authors:** Dina Yazidi, Maarten Vander Kuylen, Meriem Ennaji, Fadi Charara, Issam El Nakadi, Michel Moreau, Maria Galdon Gomez, Laurine Verset, Gabriel Liberale

**Affiliations:** 1 Department of Digestive Surgery, Institut Jules Bordet and Hôpital Erasme (HUB), Université Libre de Bruxelles (ULB), 1070 Anderlecht, Belgium; d.yazidi@gmail.com (D.Y.); maarten.vanderkuylen@hubruxelles.be (M.V.K.); meriem.ennaji@hubruxelles.be (M.E.); fadi.charara@ulb.be (F.C.); issam.elnakadi@hubruxelles.be (I.E.N.); 2 Information Management Unit, Institut Jules Bordet (HUB), Université Libre de Bruxelles (ULB), 1070 Anderlecht, Belgium; michel.moreau@hubruxelles.be; 3 Department of Pathology, Institut Jules Bordet (HUB), Université Libre de Bruxelles (ULB), 1070 Anderlecht, Belgium; maria.galdongomez@hubruxelles.be (M.G.G.); laurine.verset@hubruxelles.be (L.V.)

**Keywords:** esophagogastric junction adenocarcinoma, lymphadenectomy, right paratracheal station, postoperative complications

## Abstract

The benefit of extensive lymphadenectomy including the right paratracheal station (RPTS) in the upper mediastinum for esophagogastric junction (EGJ) adenocarcinoma remains controversial. Upper mediastinal lymph node (LN) involvement has been associated with esophageal invasion length, representing a potential research area. This study aimed to assess the rate of RPTS LN involvement in EGJ adenocarcinoma and its correlation with esophageal invasion length, as well as potential impacts on survival and postoperative complications. Patients undergoing two- or three-field esophagectomy with lymphadenectomy extended to the RPTS between 2006 and 2023 were retrospectively included. Patient, tumor, operative, and postoperative data were collected. Among 321 esophagectomies, 147 met inclusion criteria. Median esophageal invasion length was 3 cm. No patients (0%) had LN metastasis in the RPTS, regardless of invasion length (>4 cm or ≤4 cm). Postoperative complications occurred in 41.5% of patients, most commonly weight loss > 10% (29.2%), pleural effusion (21.1%), and infectious pneumonitis (19.7%). Five-year overall and disease-free survival rates were 44% and 29%, respectively. Our findings suggest that extending lymphadenectomy to the right paratracheal space fails to detect lymph node invasion in patients with esophageal invasion greater than or less than 4 cm in patients with esophageal adenocarcinoma.

## Simple Summary

Esophagogastric junction adenocarcinoma is a type of cancer occurring where the esophagus meets the stomach. Surgeons often remove nearby lymph nodes during surgery to reduce the risk of cancer spreading, but the benefit of removing nodes in the upper chest, specifically on the right side of the trachea, is unclear. In our study of 147 patients, none of these lymph nodes contained cancer, regardless of how far the tumor had spread along the esophagus. Extending the surgery to include these nodes did not appear to improve survival but was associated with typical postoperative complications such as weight loss, fluid in the lungs, and infections. These findings suggest that removing the right paratracheal lymph node may not be necessary for most patients. Future studies could focus on more precise surgical strategies to reduce complications while ensuring effective cancer treatment.

## 1. Introduction

In 2020, cancers of the esophagogastric junction (EGJ) were the seventh most common cancer type worldwide and the sixth leading cause of cancer-related death [1]. In Belgium, the incidence of esophageal cancer declined between 2007 and 2021. However, an increase in the rate of adenocarcinomas and a decrease in the rate of squamous cell carcinomas has been observed, representing 61.55% and 33.85% of cases, respectively [2]. These discrepancies can be explained by preventive measures against smoking and alcoholism, the main risk factors for squamous cell carcinoma [3], and, on the other hand, by increases in obesity and its associated complications, such as gastro-esophageal reflux disease [4], favoring the development of adenocarcinoma.

Surgery plays a central role in the treatment of early-stage adenocarcinoma of the EGJ. The type of esophageal resection performed depends on tumor location and the extent of lymphadenectomy, including either one, two, or three operative fields in the procedure. In locally advanced EGJ, perioperative chemotherapy or concomitant neoadjuvant chemoradiotherapy (NACR) followed by surgery is advised [5]. Extended lymphadenectomy in esophageal cancer improves tumor staging, reduces locoregional recurrence, and enhances survival, but is also associated with increased morbidity [4,5,6,7]. Five-year survival remains under 25%, with lymph node (LN) involvement being a key prognostic factor [8].

Kurogawa et al. have suggested that extending lymphadenectomy to the right paratracheal station (RPTS) may benefit patients with a length of esophageal invasion greater than 4 cm, and have reported a correlation between the length of esophageal tumor invasion and the rate of upper mediastinal lymph node metastases, which can reach 10.7% to 13.9% in EGJ cancers, contributing to the development of new recommendations [9,10,11].

The main objective of this study was to analyze the rate of RPTS LN involvement in patients with EGJ adenocarcinoma. Secondary objectives were to analyze correlations with esophageal length invasion (<4 cm vs. ≥4 cm) and to evaluate the impact of extending lymphadenectomy to the RPTS on survival and postoperative complications. 

This study adds to the literature by providing Western-specific data on RPTS lymph node involvement, helping to clarify the potential role of extended lymphadenectomy and guiding evidence-based surgical decision-making.

## 2. Materials and Methods

### 2.1. Patients

This was a retrospective, descriptive, bicentric study including patients with adenocarcinoma of the esophagogastric junction who underwent elective esophagectomy between 1 January 2006 and 28 February 2023. The study protocol was approved by the ethics committees of both participating hospitals, Institut J. Bordet and Erasme Hospital, Hôpitaux Universitaires de Bruxelles (HUB), Université Libre de Bruxelles (ULB) prior to data collection.

Patients with histologically proven EGJ adenocarcinoma of Siewert type I, II, or III, deemed operable and resectable by a multidisciplinary team, and older than 18 years old were included. Patients under 18 years of age, with histological types other than adenocarcinoma, and adenocarcinoma located in the middle and/or upper third of the esophagus were excluded.

### 2.2. Clinical Data and Study Objectives

Medical data were manually extracted from computerized medical records (CMR) including ‘Dossier médical informatisé’ (DMI) (Erasme hospital) and ‘Oribase’ (Institut Jules Bordet). Each record was anonymized by assigning a code to each included patient, according to the following model: a letter “E” for Erasme hospital) and “B” for Institut Jules Bordet, followed by a number.

Demographic, clinical, endoscopic, surgical, pathological, and follow-up data were collected including: age, gender, tumor size, length of esophageal invasion by tumor from Z-line and Siewert classification in endoscopy, cTNM stage, neoadjuvant treatments (e.g., chemotherapy, radiotherapy, and radiochemotherapy), tumor regression score according to Mandard or Tumor Regression Grade (TRG), presence of lymphatic, vascular, and neural invasion, type of surgery (two-way or three-way esophagectomy), pTNM stage (according to the Union for International Cancer Control TNM Classification of Malignant Tumours (7th edition), margin status, number of lymph nodes removed, adjuvant treatments, postoperative complications according to the Clavien–Dindo classification, pleural drainage, re-operation, readmission to intensive care unit (ICU), ICU stay duration, postoperative mortality at 30 days and at 90 days, disease-free survival (DFS), and overall survival (OS).

### 2.3. Surgery

In our series, the surgical approach evolved over time. Prior to 2018, patients underwent open laparotomy with thoracotomy. Between 2018 and 2022, a hybrid minimally invasive approach was used, consisting of laparoscopy for the abdominal phase and thoracotomy for the thoracic phase. Since 2022, a fully minimally invasive approach has been adopted, with laparoscopy for the abdominal phase and thoracoscopy for the thoracic phase. For three-field esophagectomy, a cervical incision was added for the cervical phase.

### 2.4. Lymphadenectomy

For lymphadenectomy, the data collected included the number of lymph nodes removed from various anatomical regions (i.e., thoracic and/or abdominal). For this study, ‘extended lymphadenectomy’ was defined as a thoracic lymphadenectomy including LNs of the RPTS, specifically lymph nodes of the 2R–4R stations as well as thoracic lymph node stations 5, 6, 7, 9, 10, and 11.

Additionally, lymph nodes from peri-esophageal station 8 were included, alongside abdominal lymph nodes from stations 1, 2, 3, 4sa, 4sb, 4d, 5, 6, 7, 8, 9, 10, 11, and 12. This space includes LN stations 2R and 4R, which correspond to the upper and lower right paratracheal space respectively.

Then, the lymph node ratio (LNR) was calculated for EGJ tumor invasion length equal to or greater than 4 cm and for invasion less than 4 cm.

### 2.5. Management of Postoperative Complications

Postoperative complications were managed according to institutional protocols and international guidelines.

Patients with weight loss of 10% or more received early assessment and dietary optimization under the supervision of a nutritionist. Pleural effusions were drained when large or symptomatic. Infectious pneumonia was treated with empirical broad-spectrum antibiotics adjusted according to culture results, combined with respiratory physiotherapy and supportive care. Anastomotic leaks were managed endoscopically by placement of a covered self-expanding metallic stent.

Patients who developed acute respiratory distress syndrome were transferred to the intensive care unit for lung-protective mechanical ventilation and advanced supportive management. Atelectasis was treated with intensive respiratory physiotherapy. Empyema was managed with targeted antibiotic therapy and pleural drainage, depending on the volume and organization of the effusion.

Chylothorax was treated with chest drainage, dietary modification to a low-fat or medium-chain triglyceride regimen, and administration of octreotide when indicated. Hemothorax was initially managed with chest tube drainage, and video-assisted thoracoscopic surgery was performed in one case.

### 2.6. Objectives

The primary objective of this study was to analyze the rate of RPTS LN involvement in patients with EGJ adenocarcinoma. Secondary objectives were to analyze correlations with esophageal invasion length (<4 cm vs. ≥4 cm), to evaluate the potential benefit of extending lymphadenectomy to the RPTS by analyzing its impact on DFS and OS, and to evaluate postoperative 30- and 90-day morbidity and mortality.

### 2.7. Statistical Analysis

Data collection and statistical analyses were carried out using Excel (Microsoft Corporation, Redmond, WA, USA) and SAS 9.4 (SAS Institute Inc., Cary, NC, USA). Data are reported as absolute values and percentages for ordinal variables. For continuous variables, the median and/or mean (with confidence interval) are reported. Descriptive statistics are used to summarize patient characteristics. Categorical variables were analyzed by the Chi2 test or Fisher’s exact test.

Continuous variables were analyzed by Student’s *t*-test or the Wilcoxon nonparametric test. Survival analysis was conducted using the Kaplan–Meier method. Univariate logistic regression analysis was used to determine independent risk factors for complications. The significance level used was 5%, i.e., *p* < 0.05.

## 3. Results

### 3.1. Study Population

Data were collected from a chart review of 321 patients for whom esophagectomy was indicated between 1 January 2006 and 31 February 2023. Among these patients, 174 patients were excluded from the study, including 130 non-adenocarcinoma patients, 35 patients who had not undergone a two- or three-way esophagectomy with RPTS lymphadenectomy, 5 patients under 18 years of age, and 4 patients who refused surgery. One hundred forty-seven patients were included in the study. Patient characteristics are reported in Table 1.

In summary, the median tumor size was 2.7 cm and the median length of esophageal invasion was 3.0 cm. Neoadjuvant treatments were administered to 118 patients (80.3%) with the most frequent treatment regimens being concomitant chemoradiotherapy for 70 patients (47.6%) and chemotherapy alone for 43 patients (29.2%). The most frequent chemotherapy regimen was 5-fluorouracil, leucovorin, oxaliplatin, and docetaxel (FLOT), administered to 40 patients. Among patients who received neoadjuvant chemotherapy, TRG was grade 1 in 15 patients (10.2%), grade 2 in 18 (12.2%), grade 3 in 26 (17.7%), grade 4 in 31 (21.1%), and grade 5 in 7 (4.8%). In 50 (34.0%) patients, TRG was not assessed. One hundred forty patients (95.2%) were treated with a two-way esophagectomy, while 7 patients had a three-way procedure (4.8%). R0 resection was achieved in 127 patients (86.4%) and 19 patients (12.9%) had an R1 resection. Lymphatic emboli were found in 56 patients (38.1%), vascular emboli in 22 (15.0%), and neural invasion in 36 (24.5%) on the operative specimen.

### 3.2. Pathology Lymph Node Staging, RPTS Status, and Outcomes

The median number of nodes removed was 26 (mean: 26.0—Range (20.0, 32.0). Sixty-nine patients (46.9%) had positive lymph nodes at final pathology, with documented involvement in specific anatomical regions. The distribution of nodal involvement according to these regions is presented in Table 2.

**Figure 1:**
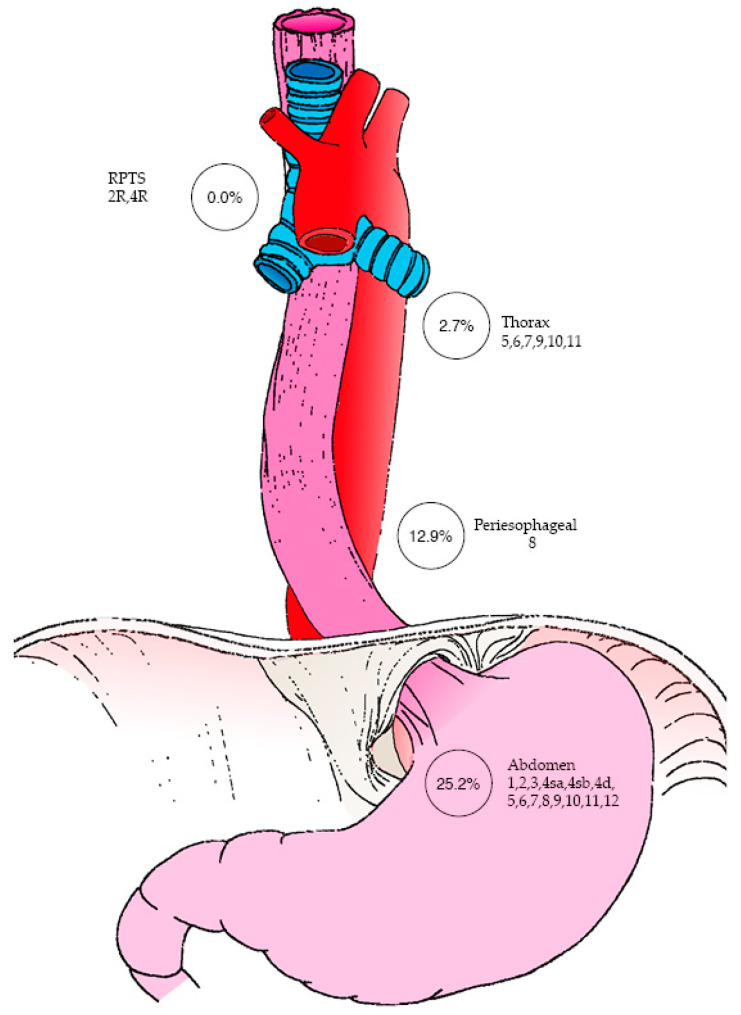
Mapping of patients with metastatic lymph nodes by anatomical region. The figure was designed by the authors, with artistic support from Timothée Défarge, inspired by the work of Kurokawa et al. [10].

Concerning RPTS LN status, none of the patients (0%) in the cohort had LN involvement on final pathology analysis. Regarding the pathological status of other LN stations (Figure 1), thoracic LNs (stations 5, 6, 7, 9, 10, and/or 11) were found to be metastatic in 14.3% of patients, with 12.9% of these cases involving peri-esophageal LNs (station 8). Additionally, abdominal LNs—including perigastric, hepatic, and splenic stations (1, 2, 3, 4sa, 4sb, 4d, 5, 6, 7, 8, 9, 10, 11 and/or 12)—were involved in 25.2% of patients.

The LNRs were 10.3% and 7.9% for esophageal involvement of <4 cm and ≥4 cm, respectively (Table 3). As none of the patients in the cohort had LN involvement of RPTS on final pathology analysis, it was impossible to set up two comparative groups (RTPS positive vs. RTPS negative) to evaluate the prognostic impact in terms of DFS and OS. 

The 5-year overall survival rates were 43% and 53%, and the 5-year disease-free survival rates were 31% and 21%, for esophageal invasion of <4 cm and ≥4 cm, respectively. 

### 3.3. Postoperative Complications

Out of 148 patients, 61 (41.5%) developed at least one complication (Table 4). Among these, 29 patients (19.7%) developed complications classified with a Clavien–Dindo score ≥ 3b: 16 patients (10.9%), 2 patients (1.4%), 2 patients (1.4%), and 9 patients (6.1%) had type 3b, 4a, 4b, and 5 complications, respectively. The most frequent complications included weight loss greater than 10% (29.2%), pleural effusion (21.1%), and infectious pneumonitis (19.7%). Other complications included anastomotic leaks (12.2%), acute respiratory distress syndromes (10.2%), atelectasis (6.8%), empyema (4.8%), chylothorax (2.0%), hemothorax (2.0%), and cardiorespiratory arrest (1.3%). No digestive duct ischemia or recurrent laryngeal nerve palsy were reported. 

Nineteen patients (12.9%) required additional pleural drainage. A total of 16 patients (10.9%) required surgical reintervention. Nine patients (6.1%) died within three months of the procedure.

**Figure 2:**
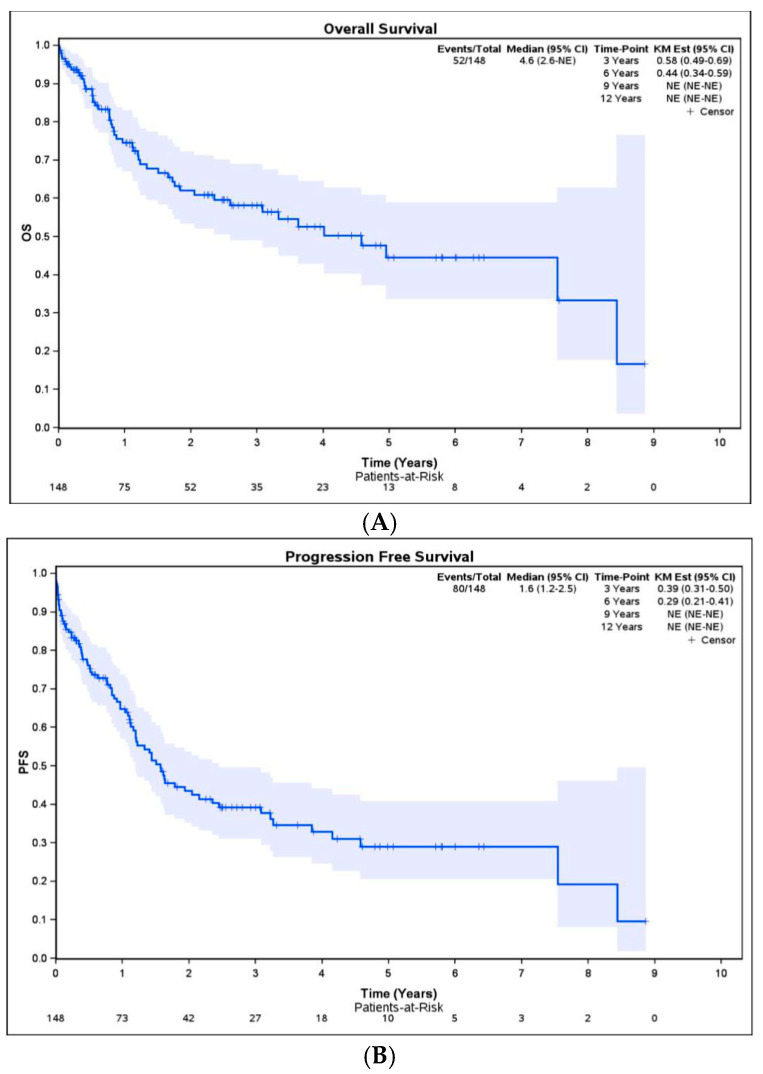
Overall survival (**A**) and disease-free survival (**B**) of patients with EGJ adenocarcinoma.

Five-year OS (Figure 2A) and DFS (Figure 2B) for the whole population with EGJ adenocarcinoma who underwent esophagectomy were 44% and 29%, respectively.

## 4. Discussion

This study aimed to evaluate the rate of RPTS LN involvement as a part of thoracic lymphadenectomy performed at our institution and to evaluate the potential impact of RPTS LN involvement in terms of outcomes in patients with EGJ adenocarcinoma treated by a two- or three-way esophagectomy. Our findings indicate that extending lymphadenectomy to the right paratracheal space does not appear to provide a significant clinical advantage, as no lymph node invasion was found in this region, regardless of the length of the EGJ invasion (<4 cm or ≥4 cm). This is in line with other Western studies reporting RPTS LN involvement in less than 5% of patients treated for EGJ or adenocarcinoma of the lower third of the esophagus [12,13].

However, these findings differ from data from Asian studies that have reported involvement of the upper mediastinal LN stations in more than 15% of patients with lower esophageal cancers [9,14,15]. More specifically, regarding LN station 2R, involvement ranging between 5% and 7% has been reported in other studies [9,16]. Conversely, the study of Yamashita et al. reported LN involvement in 0.4% of upper mediastinal stations, with no cases of involvement in station 4R, in a large cohort of patients with stage T1 and T2 EGJ adenocarcinoma [17]. These data are consistent with our findings. Unfortunately, the impact of RPTS LN involvement on survival in patients with tumors < 4 cm or ≥4 cm was not evaluable as no patients presented with LN involvement of this station. 

The postoperative complication rate in our study was 41.2%, with weight loss, pleural effusion, infectious pneumonia, anastomotic leaks, and acute respiratory distress syndrome being the most frequent complications. This is consistent with existing literature, where extended two-field lymphadenectomy is associated with higher morbidity compared to standard procedures [9,12]. The 90-day mortality rate in our cohort was 6%, slightly higher than the expected 4.5% for standard esophagectomy procedures [18]. This aligns with other studies reporting similar mortality rates after extended lymphadenectomy [12,19].

This study demonstrated a 5-year overall survival rate of 44% and a disease-free survival rate of 29%, comparable to those observed in Western populations [20]. However, these rates vary from those in Asian populations, where survival outcomes tend to be slightly higher, ranging from 51% to 53% [9,21]. While extended lymphadenectomy including RPTS LNs was associated with improved disease-free survival, it did not significantly impact overall survival in our study.

This study had several limitations. First, it had a retrospective, non-randomized design with missing data for some patients. Second, the pathological protocol lacked detailed specification of lymph node stations. Furthermore, the length of esophageal involvement was estimated from endoscopic reports, with arbitrary approximations of the Z-line when not specified, which may have led to inaccuracies, particularly in cases of circumferential tumors. 

Despite its limitations, this study offers several important strengths. It provides detailed, institution-specific data on the incidence of RPTS lymph node involvement in Western patients with EGJ adenocarcinoma—a population for which such data remain scarce. The absence of RPTS involvement across tumor lengths offers practical value for surgical planning, supporting a more selective and rational approach to lymphadenectomy. This may help limit unnecessary dissection, reduce operative time, and potentially decrease postoperative morbidity. Moreover, these findings enhance preoperative counseling by allowing clinicians to deliver individualized risk assessments.

From a surgical perspective, the study highlights the importance of targeting clinically relevant lymph node stations, thereby prioritizing oncologic benefit while minimizing procedural risk. The concordance of our results with previous Western series reinforces their external validity and supports evidence-based refinement of lymphadenectomy protocols for EGJ adenocarcinoma.

Further prospective research is warranted to better delineate the role of extended lymphadenectomy in this patient group, ideally through standardized dissection techniques and rigorous selection criteria. Integration of surgical simulation or advanced training models could facilitate the safe practice of extended lymphadenectomy and the refinement of operative skills, ultimately improving outcomes while minimizing complications.

## 5. Conclusions

This study does not allow for definitive conclusions regarding the benefit of extending lymphadenectomy to the right paratracheal space in EGJ adenocarcinomas when esophageal involvement is greater than or equal to 4 cm. The absence of LN metastasis in the right paratracheal station across the entire cohort, regardless of the length of esophageal involvement, suggests that performing a lymphadenectomy of these lymph node stations may not be crucial. 

## Figures and Tables

**Table 1:** Patient Characteristics.

	*n* = 147
Age, yr Median (range)	69.0 (64.0–75.0)
Sex Male Female	129 (87.8%) 18 (12.2%)
Tumor size *, cm Median (range)	2.7 (1.8–4.5)
Length of the esophageal involvement *, cm at endoscopy Median (range)	3.0 (1.5–5.0)
Patients with esophageal length involvement <4 cm ≥4 cm Unknown	53 (36.1%) 38 (25.9%) 56 (38.1%)
Tumor epicenter Siewert I Siewert II Siewert III Unknown	55 (37.4%) 43 (29.3%) 9 (6.1%) 40 (27.2%)
Clinical Tumor stage (cT) Tx T0 T1 T2 T3 T4	2 (1.4%) 0 (0.0%) 17 (11.6%) 29 (19.7%) 96 (65.3%) 3 (2.0%)
Clinical Node stage (cN) Nx N0 N1 N2 N3	11 (7.5%) 47 (32.0%) 74 (50.3%) 13 (8.8%) 2 (1.4%)
Clinical Metastasis stage (cM) Mx M0 M1	106 (72.1%) 41 (27.9%) 0 (0.0%)
Neoadjuvant treatment Yes No	118 (80.3%) 29 (19.7%)
TRG (patients with neoadjuvant treatment) Grade 1 Grade 2 Grade 3 Grade 4 Grade 5 Not assessed	15 (10.2%) 18 (12.2%) 26 (17.7%) 31 (21.1%) 7 (4.8%) 21 (14.3%)
Invasion at pathology Lymphatic Vascular Nerve Unknown	56 (38.1%) 22 (15.0%) 36 (24.5%) 33 (22.4%)
Surgical techniques Two-field: Laparo-thoracic approach Three-field: Laparo-thoraco-cervical approach	140 (95.2%) 7 (4.8%)
Type of resection R0 R1 R2 Unknown	127 (86.4%) 19 (12.9%) 0 (0.0%) 1 (0.7%)
Pathological T stage (pT) Tx T0 T1 T2 T3 T4	2 (1.4%) 0 (0.0%) 42 (28.6%) 24 (16.3%) 65 (44.2%) 14 (9.5%)
Pathological N stage (pN) Nx N0 N1 N2 N3	2 (1.4%) 76 (51.7%) 32 (21.8%) 19 (12.9%) 18 (12.2%)
Pathological M stage (pM) M0 ** M1	144 (98.0%) 3 (2.0%)
Number of retrieved Lymph Nodes on operative specimen * Median (range)	26.0 (20.0–32.0)
Adjuvant treatment Yes No Unknown	57 (38.8%) 74 (50.3%) 16 (10.9%)

* Includes missing data. ** Preoperative negative staging.

**Table 2:** Lymph node status of retrieved LNs according to anatomical region per patient.

	Total Patients *n* = 147	pN0 *n* = 77 (52.4%)	pN+ *n* = 70 (47.6%)
**Specified LN stations in pathology report**	145 (98.6%)	75 (51.0%)	70 (47.6%)
***Thoracic nodes***			
Subaortic (st. 5) Para-aortic (st. 4) Subcarinal (st. 7) Triangular ligament (st. 9) Hilar (st. 10) Interlobar (st. 11)		125 (85.0%)	4 (2.7%)
Peri-esophagus (st. 8)		64 (43.5%)	19 (12.9%)
RPTS (st 2R, 4R)		108 (73.5%)	0 (0.0%)
***Abdominal nodes***			
Perigastric(st. 1, 2, 3, 4sa, 4sb, 4d, 5, 6, 7) Common-hepatic artery (st. 8) Celiac (st. 9) Splenic hilum and splenic-artery (st. 10,11) Hepatoduodenal ligament (st. 12)		51 (34.7%)	37 (25.2%)
**Unspecified LN stations in pathology report**	2 (1.36%)	2 (1.36%)	0 (0.0%)

LN: lymph node; pN0: no lymph node involvement at pathology; pN+: lymph node involvement at pathology; RTPS: right paratracheal station; st: station.

**Table 3:** Lymph node ratio (LNR) as a function of the length of esophageal invasion.

	Esophageal Invasion Length < 4 cm (*n* = 53)	Esophageal Invasion Length ≥ 4 cm (*n* = 38)	Missing or Uncertain Data (*n* = 56)
Total lymph nodes *	26.5	26.2	26.1
Metastatic lymph nodes * mean	2.7	2.1	2.6
**LNR**	**10.3%**	**7.9%**	**10%**
5-year OS	43%	53%	40%
5-year DFS	31%	21%	17%
5-year OS	44%		
5-year DFS	29%		

DFS: Disease-free survival; LNR: Lymph node ratio; OS: Overall survival. * Includes missing data.

**Table 4:** Postoperative complications following esophagectomy with extended two-field lymphadenectomy.

	*n* = 147
Postoperative Complications Yes No Type of postoperative complication Weight loss ≥ 10% Pleural effusion Infectious pneumonia Anastomotic leak Acute respiratory distress syndrome Atelectasis Empyema Chylothorax Hemothorax Cardiac arrest Conduit ischemia Recurrent laryngeal nerve paralysis	61 (41.5%) 86 (58.5%) 43 (29.2%) 31 (21.1%) 29 (19.7%) 18 (12.2%) 15 (10.2%) 10 (6.8%) 7 (4.8%) 3 (2.0%) 3 (2.0%) 2 (1.4%) 0 (0.0%) 0 (0.0%)
Clavien–Dindo Classification 1 2 3a 3b 4a 4b 5	8 (5.4%) 11 (7.5%) 13 (8.8%) 16 (10.9%) 2 (1.4%) 2 (1.4%) 9 (6.1%)
Pleural drainage	19 (12.9%)
Surgical reintervention Yes No	16 (10.9%) 131 (89.1%)
Readmission to ICU Yes No	19 (12.9%) 128 (87.1%)
CU stay duration (days), Median	1.2 (0–34)
30-day mortality	5 (3.4%)
90-day mortality	9 (6.1%)

## Data Availability

The data presented in this study are available on request from the corresponding author. Due to the sensitive nature of patient information and the ethical approval terms of the parent study, the data cannot be shared publicly. Access to the data requires approval from the ethics committee and compliance with data protection regulations.
